# Expression of TCF7L2 in Glioma and Its Relationship With Clinicopathological Characteristics and Patient Overall Survival

**DOI:** 10.3389/fneur.2021.627431

**Published:** 2021-07-08

**Authors:** Shiyuan Jing, Lei Chen, Song Han, Ning Liu, MingYang Han, Yakun Yang, Changxiang Yan

**Affiliations:** Sanbo Brain Hospital, Capital Medical University, Beijing, China

**Keywords:** glioma, TCF7L2, overall survival, prognosis, clinicopathological characteristics

## Abstract

**Background:** The TCF7L2 gene is known as transcription factor 7-like 2 which has been identified as a novel transcription factor epithelial-mesenchymal transition (EMT) in tumor cells at 10q25.3. TCF7L2 may affect cancer progression and plays a central role in cancer proliferation, migration, and invasion. However, its clinical and prognostic value have not been researched in glioma. The purpose of our study was to research TCF7L2 expression and evaluate the clinical value of prognosis.

**Method:** We collected glioma specimens including low-grade glioma (*n* = 46) and glioblastoma (*n* = 51) from September 2015 to September 2017. Expression of TCF7L2 in 97 specimens was detected by quantitative real-time PCR (qRT-PCR). The chi-square test was applied to analyze the relationship between TCF7L2 expression and clinicopathological characteristics. The overall survival (OS) was estimated by log-rank tests among strata, and the survival curves were drawn by Kaplan-Meier. Univariate and multivariate analysis were utilized to analyze the relationship between prognosis and clinicopathological characteristics including TCF7L2 expression.

**Results:** Compared with the low-grade glioma group, the expression of TCF7L2 was significantly increased in the glioblastoma group (*p* = 0.001). TCF7L2 overexpression was associated with higher WHO grade (*p* = 0.001), isocitrate dehydrogenase (IDH) wild-type (*p* = 0.001), and lack of O(6)-methylguanine-DNA methyltransferase (MGMT) methylation (*p* = 0.001). Moreover, Kaplan-Meier analysis proved that overexpressed TCF7L2 was associated with poor OS (*p* = 0.010). The multivariate analysis suggested that TCF7L2 expression was an independent prognostic factor (*p* = 0.020).

**Conclusions:** Our research proved that TCF7L2 was overexpressed in glioblastoma, and related with tumor long-term prognosis, which, therefore, could be an independent prognostic factor for glioma patients.

## Background

Glioblastoma is a common malignant brain tumor owing to its stronger invasive ability and resistance to treatment. The morbidity rate of glioblastoma is approximately 0.59–0.69/100000 people worldwide, affecting those aged 20 years or older, especially those aged 40–70 years (1). The morbidity is higher in men (3.97/100,000) than in women (2, 3) (2.53/100,000).

According to NCCN Guidelines, the standard therapies include surgery, radiotherapy with concomitant temozolomide (TMZ), adjuvant TMZ chemotherapy, and TTF therapies, but the 5-year overall survival (OS) is only 9.8% (3). Despite comprehensive therapy, the median survival time is ~12–15 months for GBM patients after diagnosis (4, 5).

An increasing number of molecular markers have been discovered, which improves our understanding of the mechanism of glioma and development. To date, the final histological diagnosis, final integrated diagnosis, pathological classification, and prognosis assessment are more accurate, which can help develop personalized therapy for glioma. Therefore, it is important to find novel biomarkers which can predict the prognosis, and explore its potential as a therapeutic target for glioma patients.

The Wnt/β-catenin signaling pathway is important in the majority of tumor progression. There is over 90% of aberrant activation of Wnt signaling in colorectal cancer (6). TCF7L2 is a member of the Wnt/β-catenin signaling pathway, which plays an important role in metabolism, cell differentiation/ proliferation, and cell death (7). Several meta-analyses have evaluated cancer risk induced by TCF7L2 gene variants (8–10).

A study (11) provided summary evidence that TCF7L2 genes are associated with the risk of breast, colorectal, and lung cancer and glioma. The loss of TCF7L2 enhances tumor cell growth, whereas a gain inhibits tumor cell growth (12). TCF7L2 plays a significant role in the pathogenesis of human cancers. A recent study (13) demonstrated that LEF/TCF-specific transcriptional regulation of Wnt target genes is associated with cancer progression and survival in human colorectal tumor samples.

However, the study of TCF7L2 mutation mainly focused on breast, colorectal, and lung cancer. Therefore, the aim of our study was to analyze the clinical value of TCF7L2 in glioma. We collected samples including 51 GBM tissues and 46 low-grade glioma tissues WHO (I-II). We focused on the TCF7L2 effect and the expression level in glioma tissue. Subsequently, we analyzed the relation between TCF7L2 expression level and clinicopathological characteristics. In our study, we performed a preliminary analysis between the expression level of TCF7L2 and overall survival risk of glioma among Chinese people.

## Methods

### Patients and Tissue Samples

Patients who underwent initial surgery in the Sanbo Brain Hospital of Capital Medical University from September 2015 to September 2017 were retrospectively selected for this research. All samples were diagnosed by pathologists and stored in liquid nitrogen.

As for patients with low-grade glioma, we identified high-risk patients according to the literature (14), including at least three factors (age >40 years old, astrocytomas, tumor maximum diameter ≧6 cm, tumors across the midline, and preoperative neurological impairment). Those patients were treated with radiochemotherapy, while the patients at low risk were followed up. Patients with glioblastoma received radiotherapy with concomitant temozolomide (TMZ) and adjuvant TMZ chemotherapy.

The patients' characteristics are described in Table 1. Tissue sample usage was approved by Ethics Committee of the Sanbo Brain Hospital of Capital Medical University and written informed consent was obtained from all study participants.

**Table 1:** Relationship between the expression level of TCF7L2 and clinicopathological parameters.

**Characteristic**	**Case number**	**TCF7L2 expression** ***R***_****s****_		***p***
		**Low (*n* = 47)**	**High (*n* = 50)**	
Age (years)				0.980
≤45	35	17	18	
>45	62	30	32	
Gender				0.970
Male	60	29	31	
Female	37	18	19	
Tumor size (cm)				0.085
≤3	33	20	13	
>3	64	27	37	
Necrosis				0.618
Yes	47	24	23	
No	50	23	27	
WHO grade			0.41	0.001
I-II	46	33	13	
IV	51	14	37	
IDH status			0.32	0.001
Wild-type	54	18	36	
Mutation	43	29	14	
1p/19q status				0.180
No deletion	72	32	40	
Co-deletion	25	15	10	
MGMT status			0.37	0.001
No methylation	55	17	38	
Methylation	42	30	12	

### RNA Extraction and qRT-PCR Analyses

The total RNA was extracted from the frozen sample by TRIzol reagent (Invitrogen, Carlsbad, CA, USA). RNA was reverse-transcribed to complementary DNA (cDNA) using a PrimeScript RT reagent kit (Thermo, Beijing, China) according to the manufacturer's protocol. Its synthesis was conducted at 37°C for 15 min, then 85°C for 5 s according to the experimental protocols. Real-time PCR reactions were carried by Applied Biosystems 7500. Real-time PCR was carried in triplicate. We selected glyceraldehyde3-phosphate dehydrogenase (GAPDH) as a suitable endogenous reference gene. The relative TCF7L2 expression was computed and normalized using the 2-^ΔCT^ method relative to GAPDH. The primers for TCF7L2: 5′-TGCTCTGCGGTTGCTATGTTGAC-3′, 3′-GCT GCGAGTCCTCACCAATGTC-5′ and for GAPDH: 5′-CAGACCACAG TCCATGCCATCAC-3′, 3′-GACGCCTGCTTCACCACCTTC-5′.

### Data Analysis

The comparison of the TCF7L2 expression level between GBM and low-grade glioma was performed by the two-sample Student's *t*-test. The chi-square test was used to examine the associations between TCF7L2 expression and the clinicopathological characteristics.

In addition, survival curves were drawn by the Kaplan-Meier method and analyzed with log-rank test. Cox proportional-hazards regression analysis was applied to estimate univariate and multivariate hazard ratios for OS. A value of *P* < 0.05 was considered as statistically significant. SPSS software 20.0 was applied in the present study.

## Results

### TCF7L2 Was Upregulated in Glioblastoma Tissues

**Figure 1:**
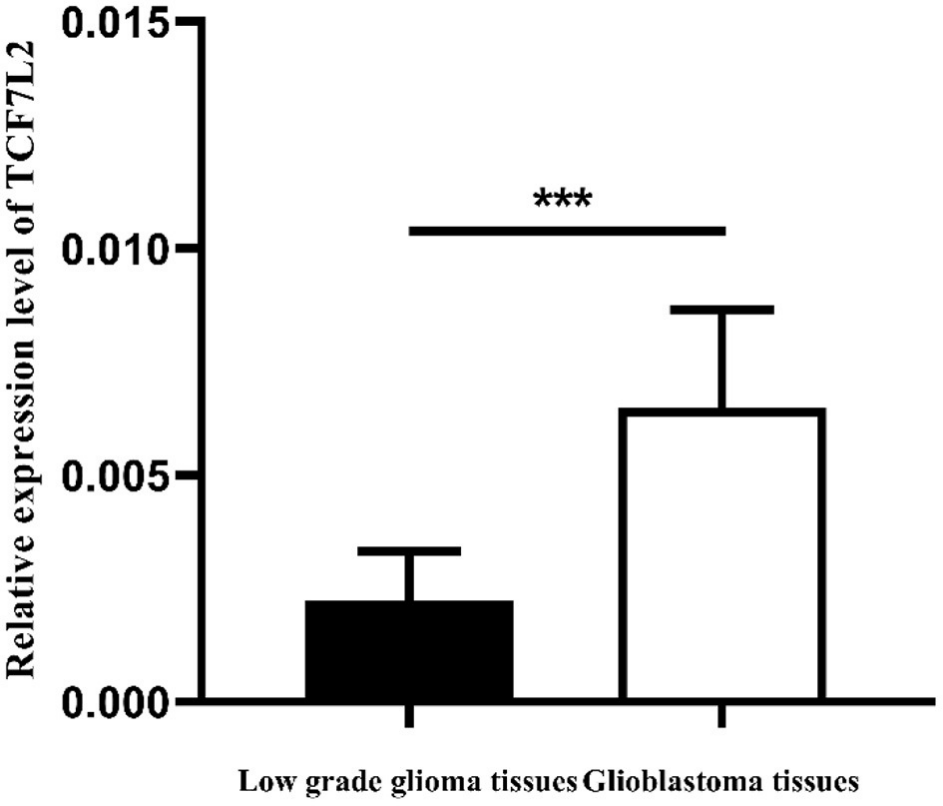
Expression levels of TCF7L2 in GBM tissues were significantly higher (***p = 0.001).

We measured TCF7L2 expression levels in 97 patients with glioma by qRT-PCR. The data revealed that TCF7L2 had a higher expression in GBM tissues than the low-grade group (*p* = 0.001) (Figure 1).

### Relation Between TCF7L2 Expression and Clinicopathological Factors of GBM Patients

We found high TCF7L2 expression in GBM. The median expression level of TCF7L2 was used as a dividing point, and we divided 97 patients into two groups (high and low expression). Table 1 summarizes the relationship between TCF7L2 expression and clinicopathological parameters in glioma. The results showed that TCF7L2 high expression was significantly related to higher WHO grade (*p* = 0.001), isocitrate dehydrogenase (IDH) wild-type (*p* = 0.001), and lack of O(6)-methylguanine-DNA methyltransferase (MGMT) methylation (*p* = 0.001).

### Upregulation of TCF7L2 Confers Poor Prognosis in Patients

Univariate and multivariate analyses were utilized to evaluate the association between OS and various clinicopathological features including TCF7L2 expression level (Table 1).

**Figure 2:**
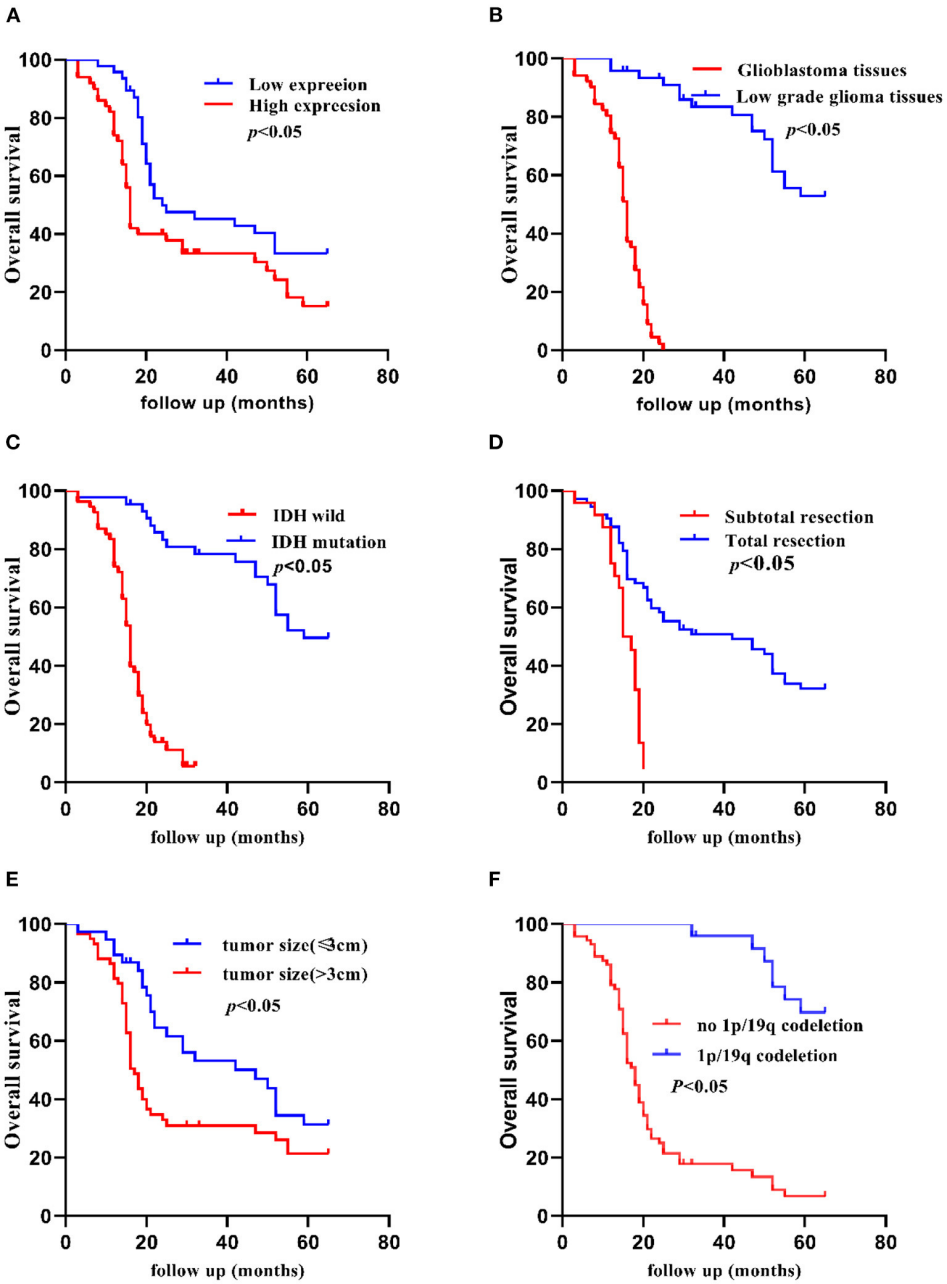
Kaplan-Meier 5-year OS curves of patients with glioma according to clinicopathological features. **(A)** Patients with higher TCF7L2 expression showed shorter OS compared with those with a lower expression (*p* = 0.010), **(B)** Glioblastoma patients showed worse OS compared with low-grade patients (*p* = 0.001). **(C)** Patients with IDH wild-type showed worse OS compared with those with an IDH mutation (*p* = 0.010). **(D)** Patients who had a subtotal resection showed worse OS compared with those who had a total resection (*p* = 0.010). **(E)** Patients with a larger tumor size (>3 cm) showed worse OS compared with those with a small tumor size (≤3 cm) (*p* = 0.020). **(F)** Patients with no 1p/19q codeletion showed worse OS compared with those with 1p/19q codeletion (*p* = 0.001).

**Figure 3:**
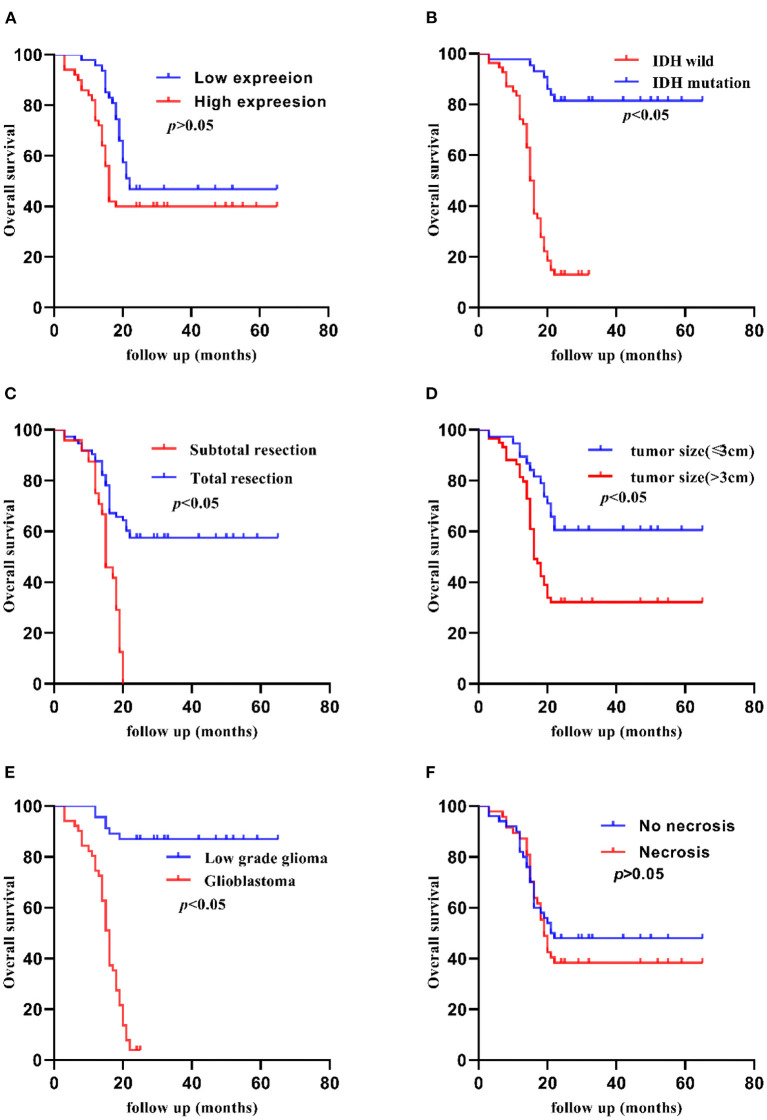
Kaplan-Meier 2-year OS curves of patients with glioma according to clinicopathological features. **(A)** Patients with higher TCF7L2 expression showed no significant differences compared with those with a lower expression (*p* = 0.008). **(B)** Patients with IDH wild-type showed worse OS compared with those with an IDH mutation (*p* = 0.001). **(C)** Patients who had a subtotal resection showed worse OS compared with those who had a total resection (*p* = 0.001). **(D)** Patients with a larger tumor size (>3 cm) showed worse OS compared with those with a small tumor size (≤3 cm) (*p* = 0.003). **(E)** Glioblastoma patients showed worse OS compared with low-grade patients (*p* = 0.001). **(F)** Patients with necrosis showed no significant differences compared with those with no necrosis (*p* = 0.486).

The Kaplan-Meier method indicated that the 5-year OS of patients was significantly shorter in patients with high TCF7L2 expression than in those with low TCF7L2 expression (*p* = 0.010), but there were no significant differences in 2-year OS (*p* = 0.070). The OS was significantly longer with small tumor size (≦3 cm) compared to those with large tumor size (>3 cm) (*p* = 0.020). The OS was significantly shorter in patients with IDH wild-type than in those with IDH mutation (*p* = 0.010). The OS was significantly shorter in patients receiving subtotal resection than those receiving total resection (*p* = 0.010), but was significantly longer in patients in the low-grade group compared to those with glioblastoma (*p* = 0.001). The OS was significantly shorter in patients with no 1p/19q deletion than in those with 1p/19q codeletion (*p* = 0.001). The OS was significantly shorter in patients with no MGMT methylation than in those with MGMT methylation (*p* = 0.010). There were no significant differences between necrosis and non-necrosis patients (*p* = 0.420) (Figures 2, 3).

As for multivariate Cox regression analysis including TCF7L2 expression, WHO grade, extent of resection, 1p/19q status, IDH status, and MGMT status, we found a 5-year OS advantage of the low expression vs high expression of TCF7L2 (HR 2.56, 95% CI:1.16–5.68, *p* = 0.020), low-grade glioma vs. glioblastoma (HR 79.94, 95% CI:18.88–338.39, *p* = 0.001), total resection vs subtotal resection (HR 2.09, 95% CI:1.09–4.01, *p* = 0.030), IDH mutation vs IDH wild-type (HR 0.36, 95% CI:0.15–0.87, *p* = 0.020), 1p/19q codeletion vs no 1p/19q deletion (HR 0.33, 95% CI: 0.12–0.93, *p* = 0.040), and MGMT methylation vs no MGMT methylation (HR 0.34, 95% CI:0.16–0.71, *p* = 0.010) (Table 2).

**Table 2:** Univariate and multivariate analyses of 5-year OS.

**Parameter**	**Univariate**			**Multivariate**		
	**HR**	**95% CI**	***P***	**HR**	**95% CI**	***P***
Age (≤45/>45)	1.01	1 0.61–1.67	0.960			
Sex (Male/female)	0.85	0.52–1.38	0.510			
Tumor size (≤3 cm/>3 cm)	1.77	1.07–2.92	0.020			
Necrosis (Yes/no)	1.21	0.75–1.95	0.430			
WHO grade (I-II/IV)	38.64	12.84–116.24	0.001	79.94	18.88–338.39	0.001
TCF7L2 expression (High/low)	1.86	1.14–3.01	0.010	2.56	1.16–5.68	0.020
IDH status (Wild-type/mutation)	0.09	0.04–0.19	0.010	0.36	0.15–0.87	0.020
Extent of resection (Subtotal/total)	6.12	3.36–11.17	0.010	2.09	1.09–4.01	0.030
1p/19q status (No deletion/co-deletion	0.10	0.05–0.23	0.001	0.33	0.12–0.93	0.040
MGMT status (No methylation/methylation	0.53	0.32–0.85	0.010	0.34	0.16–0.71	0.010

## Discussion

TCF7L2 has been reported to have an effect on the suppression of cell proliferation, migration, and invasion in the literature (15). The transcription factor 7-like 2 (TCF7L2) gene may affect cancer development and prognosis because the TCF7L2 gene plays an important role in the Wnt/β-catenin signaling pathway (16, 17).

To date, various biological markers have been reported in glioma (18, 19). TCF7L2 represents a central factor in metabolism, cell proliferation, and cell apoptosis (20, 21). Bo Yu et al. (15) found that TCF7L2 overexpression increased cell viability, migration, and invasion in cells with CRNDE inhibition. The TCF7L2 expression and prognostic value in glioma have rarely been reported.

In our study, we detected significantly higher TCF7L2 expression in GBM tissues than in the low-grade group. Moreover, TCF7L2 overexpression was significantly related with higher WHO grade, IDH wild-type, and no MGMT methylation, and the coefficients were 0.41, 0.32, and 0.37, respectively.

Our study reported shorter OS in patients with higher TCF7L2 expression, however, there was no significant difference in 2-year OS in the two groups. We found that the 2-year OS was relatively high in the patients who underwent standard therapies including radiotherapy with concomitant temozolomide (TMZ) and adjuvant TMZ after surgery in the high expression group. There was statistical significance in 5-year survival rate. On the other hand, TCF7L2 might be more advantageous in judging long-term prognosis.

Multivariate analysis revealed that TCF7L2, WHO grade, IDH status, extent of resection, 1p/19q status, and MGMT methylation status were independent prognostic factors for OS. The hazard ratio in the high TCF7L2 expression group was 2.56 times more than the low expression group (95% CI: 1.16–5.68, *p* = 0.020). TCF7L2 expression was independently related with OS, indicating that higher TCF7L2 level was a marker of poor prognosis for patients.

IDH mutation was found in both low-grade glioma and glioblastoma in our study, suggesting the IDH gene played an important role in the pathogenesis of tumors in glioma. The mutation rate was 15.6%, which was practically consistent with that reported in the literature (22). The multivariate analysis revealed that the patients of IDH mutation were closely associated with better prognosis, as compared with those of IDH wild-type.

Previous studies have reported that IDH mutations have been identified as one of the most important diagnostic and prognostic factors of gliomas (23). Due to intra-group heterogeneity, we need additional prognostic factors to subdivide the prognosis results in gliomas. There was a correlation between IDH status and TCF7L2 expression level, therefore, we would combine IDH status and TCF7L2 expression to further refine the stratified study and better judge the prognosis in a future study.

Previous studies have reported that patients with 1p/19q co-deletion have better prognosis and response to treatment (24, 25). 1p/19q co-deletion is a typical molecular genetic feature of oligodendroglioma, which provides an important reference for pathological diagnosis. We found that patients with 1p/19q co-deletion had a better prognosis than those no 1p/19q co-deletion. The results were consistent with literature reports.

Surgical resection plays a critical role in glioma therapy, which can reduce tumor load and provide an opportunity for postoperative adjuvant therapy. We found that patients receiving gross total resection had a better prognosis than those receiving subtotal resection. The results were consistent with other literature reports (26).

Glioblastoma patients showed worse OS compared with low-grade patients. In our study, we did not include WHO III patients, therefore, the hazard ratio was larger in the multivariate analysis. In addition, the sample size of data was relatively small, which might have introduced a bias. Currently, MGMT methylation is a widely accepted biomarker in glioblastoma, which can predict the effect of chemotherapeutic drugs (27). Patients with MGMT methylation showed a better prognosis than those with no methylation.

The sample size of data was relatively small. Some patients were reluctant to attend follow-up appointments, or the follow-up was interrupted in our study. We will increase the sample size for detailed study in the future.

TCF7L2 could be a potential prognostic factor and therapeutic target for patients with glioma. The underlying molecular mechanisms of TCF7L2 involvement in the Wnt/β-catenin signaling pathway needs to be investigated in future studies.

## Data Availability Statement

The raw data supporting the conclusions of this article will be made available by the authors, without undue reservation.

## Ethics Statement

The studies involving human participants were reviewed and approved by the Capital Medical University Hospital Ethics Committees. The patients/participants provided their written informed consent to participate in this study.

## Author Contributions

LC, SH, and NL analyzed and interpreted the glioma patient data. SJ performed the qRT-PCR of the glioma tissues. CY and SJ were major contributors in writing the manuscript. All authors read and approved the final manuscript.

## Conflict of Interest

The authors declare that the research was conducted in the absence of any commercial or financial relationships that could be construed as a potential conflict of interest. The Reviewer YW declared a shared affiliation, with no collaboration, with the authors SJ, LC, SH, NL, MH, YY, and CY.

## Abbreviations

TCF7L2: Transcription factor 7-like 2
qRT-PCR: Real-time quantitative PCR
OS: Overall survival
TMZ: Temozolomide
EMT: Epithelial-mesenchymal transition
TTF: Tumor treating fields
GBM: Glioblastoma
cDNA: Complementary DNA
HR: Hazard ratios
CI: Confidence interval
MGMT: O(6)-methylguanine-DNA methyltransferase
IDH: Isocitrate dehydrogenase.
